# Kinetics Drying of Blackberry Bagasse and Degradation of Anthocyanins and Bioactive Properties

**DOI:** 10.3390/antiox10040548

**Published:** 2021-04-01

**Authors:** Dorila E. Grández-Yoplac, Diner Mori-Mestanza, Lucas D. Muñóz-Astecker, Ilse S. Cayo-Colca, Efraín M. Castro-Alayo

**Affiliations:** 1 Programa Académico de Ingeniería Agroindustrial, Facultad de Ingeniería y Ciencias Agrarias, Universidad Nacional Toribio Rodríguez de Mendoza de Amazonas, Calle Higos Urco N° 342-350-356, Chachapoyas 01001, Amazonas, Peru; yoplacesteffany@gmail.com; 2 Instituto de Investigación, Innovación y Desarrollo para el Sector Agrario y Agroindustrial de la región Amazonas (IIDAA), Facultad de Ingeniería y Ciencias Agrarias, Universidad Nacional Toribio Rodríguez de Mendoza de Amazonas, Calle Higos Urco No. 342-350-356, Chachapoyas 01001, Amazonas, Peru; diner.mori@untrm.edu.pe (D.M.-M.); lucas.munoz@untrm.edu.pe (L.D.M.-A.); 3 Facultad de Ingeniería Zootecnista, Agronegocios y Biotecnología, Universidad Nacional Toribio Rodríguez de Mendoza de Amazonas, Calle Higos Urco 342-350-356, Chachapoyas 01001, Amazonas, Peru; icayo.fizab@untrm.edu.pe

**Keywords:** drying process, blackberry bagasse, anthocyanins, kinetics, total phenolic content, antioxidant capacity

## Abstract

The process of drying food is necessary to preserve it; however, some bioactive compounds can be degraded during drying process. In this work, the convective drying process of Peruvian blackberry bagasse and the degradation of anthocyanins, total phenolic content (TPC), and antioxidant capacity (AC) were studied. The logarithmic model fitted well to the data and could predict the process, showing that 6 h of drying at 90 °C is enough to reach equilibrium moisture. Anthocyanin degradation followed a first-order kinetic model with reaction rate constant between 5.45 × 10^−2^ ± 4.68 × 10^−3^ and 1.21 × 10^−1^ ± 2.31 × 10^−2^ h^−1^, and activation energy of 25.11 kJ/mol. The highest retention (84.38%) of anthocyanins was obtained in 1 h at 50 °C and the highest degradation (68.54%) in 6 h at 90 °C. The TPC and AC increased with the drying time and temperature due to the increased water evaporation.

## 1. Introduction

The revalorization of agrifood waste has become an important topic [1]. Consumers’ attitudes toward a healthy diet rich in natural bioactive compounds led to the search for bioactive compounds retained in the agrifood [2]. It was reported that blackberry is high in polyphenols [3], particularly anthocyanins, which are suggested to play a significant role in improving antioxidant defense mechanisms in vitro and in vivo [4]. Anthocyanins’ chemical stability is the main focus of many recent studies due to their beneficial health effects [5,6]. However, anthocyanins’ stability is affected by several factors such as temperature [5,7]. Additionally, food processing generally involves thermal treatments; hence, evaluating the heat-induced anthocyanin degradation is of utmost importance for the food industry [5]. Drying is an important processing step in ensuring product stability in terms of shelf life and quality maintenance for the utilization as a value-added product [8].

Extended shelf life and convenient industrial application have led to the increasingly widespread use of drying technology in fruit and vegetable processing. However, the drying process usually damages the active ingredients in fruits and vegetables [9]. Therefore, the drying process’s mathematical modeling is required to control the process in terms of identifying suitable operating parameters for minimizing drying time and ensuring optimal product quality [8].

The kinetic study of food drying processes is based on studying the temporal evolution of foods’ moisture content [10]. In the work of Blank et al. [11], the *Bunchosia glandulífera* pulp drying curves were validated using Page’s mathematical model. Bioactive compounds were the most sensitive to high temperatures, and the antioxidant activity was mainly affected by the drying time. In the work of López-Vidaña et al. [12] on blackberries, the results show that the Midilli–Kuçuk model provides the best fit to the experimental data of solar drying. In the study made by Martín-Gómez et al. [10] on blueberry convective drying-process at 30, 40, and 50 °C, the increase in temperature incremented the total phenolic content (TPC), the anthocyanin concentration, and the antioxidant capacity (AC). Costa et al. [13] reported that the anthocyanin thermal degradation of açai pulp through a temperature range from 40 °C to 80 °C, resulted in activation energy (*Ea*) of 24.16 kJ/mol and half-life times between 10.7 h to 28.6 h.

Berry bagasse is a natural resource of phytochemicals for developing food additives or nutritional supplement applications [14]. These phytochemicals can be affected by the agroindustrial process conditions such as drying, for which their characterization is necessary. In this sense, the present work aims to characterize the drying process’s kinetics and the degradation of anthocyanins and bioactive blackberry bagasse properties.

## 2. Materials and Methods

### 2.1. Materials

Mature and healthy fruits of blackberry (*Rubus roseus* Poir) were harvested from María town (Amazonas Region). One kilogram of fruit was stored at −20 °C until its chemical analysis.

### 2.2. Drying Process of Blackberry Bagasse

Twenty grams of blackberry bagasse were exposed to different times of drying process. According to [11], the sample was heated for specific periods of time, up to 9 h, in a sterilizing oven at different temperatures of 50, 60, 70, 80, and 90 °C. The moisture was measured with a moisture analyzer (Mettler Toledo, Excellence Plus HX204, Greifensee, Switzerland). All moisture results were expressed on a dry basis (g water/g dry weight) [15]. Water content analyses were performed in quadruplicate. According to Méndez-Lagunas et al. [16], the dry solids (DS) content, expressed as grams of dry weight (gdw), was calculated using Equation (1), as follows:

(1) $$DS=W_{t}\left(1-X_{wb}\right)$$

where $X_{wb}$ is the water content, wet basis (g water/gfw) at time t, and $W_{t}$ is the sample weight (g) at time t.

### 2.3. Anthocyanin Quantification

According to Jiang et al. [17], the monomeric anthocyanin content was determined using the pH differential method with some modification. Briefly, an aliquot (0.3 mL) of the sample was mixed with pH 1.0 (potassium chloride buffer, 9.7 mL) and pH 4.5 (sodium acetate buffer, 9.7 mL) solutions, respectively, and equilibrated for 30 min at room temperature in the dark. A visible spectrophotometer (Unico, S2100, Dayton, NJ, USA) was used to measure the absorbance at 510 nm and 700 nm [18], using water as a reference. The total anthocyanin content was calculated as cyanidin-3-glucoside (c-3-g) equivalent by the following Equation (2):

(2) $$Total\;anthocyanins\left(mg/L\right)=A\times MW\times DF\times 1000/\left(\varepsilon \times 1\right)$$

where $A=\left[{\left(A_{510}-A_{700}\right)}_{pH1.0}-{\left(A_{510}-A_{700}\right)}_{pH4.5}\right]$; MW (molecular weight) = 449.2 g/mol for c-3-g; DF = dilution factor (9.7/0.3) [19]; 1 = pathlength in cm; ε = 26,900 molar extinction coefficient in L/mol/cm for c-3-g; 1000 = conversion from g to mg. All analyses were done in quadruplicate (*n* = 4).

The effect of drying condition on the anthocyanin concentration was expressed in units of dry weight (gdw) using Equation (3), as follows:

(3) $$Anthocyanin\;concentration\left[\frac{mg}{g_{dw}}\right]=\frac{C_{ea}\times V_{s}}{1000\times DS}$$

where $C_{ea}$ is the anthocyanin concentration (mg/L), $V_{s}$ is the volume of the solution used for extraction (mL), and DS (gdw) is the dried solids in the sample used to measure the concentration calculated according to Equation (1) [16].

### 2.4. Determination of Total Phenolic Content

TPC in aqueous extracts was determined according to the Folin–Ciocalteu’s procedure [20,21]. Briefly, 0.05 mL of diluted extract and 0.45 mL water were mixed with 2.5 mL of 1:10 diluted Folin-Ciocalteu’s phenol reagent, followed by 2 mL of 7.5% (*w/v*) sodium carbonate. After 5 min at 50 °C, absorbance was measured at 760 nm using a UV/Visible spectrophotometer (Unico, S2100, Dayton, NJ, USA). TPC was estimated from a standard curve of gallic acid (*y* = 0.0447*x* + 0.0381, *R*^2^ = 0.9953). The TPC was expressed on a wet basis as mg gallic acid equivalents (GAE)/100 gfw [22] to demonstrate the concentration-effect resulting from water evaporation.

### 2.5. Antioxidant Capacity

According to Moura et al. [23], the AC was estimated from a Trolox standard curve for which Trolox standard solutions (2000 µM) were prepared in 10 mL volumetric flasks, varying the concentration from 100 µM to 1500 µM. Trolox concentrations (µM) were plotted. A 30 µL aliquot of the berry pulp or bagasse extract was mixed with 3 mL of the DPPH solution and homogenized. Then it was placed for 30 min at room temperature in the dark. Next, ethyl alcohol was used as a blank to calibrate the spectrophotometer (Unico, S2100, Dayton, NJ, USA). The reading was done at 517 nm. The results of antioxidant capacity (AC) were expressed as µM Trolox/gfw to confirm the results of Martín-Gómez et al. [22].

### 2.6. Mathematical Modeling of the Blackberry Bagasse Drying Kinetics

According to Martín-Gómez et al. [22], different mathematical models commonly used for drying processes (Equations (4)–(8)) were adjusted using the “Seed Water” package developed by Silva [24] in RStudio software. In these models, MR represents the moisture ratio measured during the drying process, and k is the reaction rate constant, t is the time of drying.

(4) $$\mathrm{Page}\text{:}\;\;\;\;\;\;\;\;\;\;MR=exp\left(-kt^{n}\right)$$

(5) $$\mathrm{Henderson}\;\mathrm{and}\;\mathrm{Pabis}\text{:}\;\;\;MR=aexp\left(-kt\right)\;$$

(6) $$\mathrm{Newton}\text{:}\;\;\;\;\;\;\;\;\;\;\;\;\;\;\;\;\;\;\;\;\;\;\;\;MR=exp\left(-kt\right)\;$$

(7) $$\mathrm{Logarithmic}\text{:}\;\;\;\;\;\;\;\;\;\;\;\;\;\;\;\;\;\;\;\;\;\;\;\;\;\;\;\;MR=a\;\times exp\left(-kt\right)+b\;$$

(8) $$\mathrm{Wang}\;\mathrm{and}\;\mathrm{Singh}\text{:}\;\;\;\;\;\;\;\;\;\;\;\;\;\;\;MR=1+at+bt^{2}\;$$

In the “Seed Water” package, MR is automatically calculated through the Equation (9):

(9) $$MR=\frac{M_{t}-M_{f}}{M_{i}-M_{f}}$$

where $M_{t}$ represents the moisture content at any time of drying, $M_{i}$ is the initial moisture content, and $M_{f}$ is the equilibrium moisture content, all on a dry basis (g water/gdw) [24]. According to Martín-Gómez et al. [22], the moisture content at equilibrium is relatively small to the others; it is assumed that Equation (10) can be simplified as follows:

(10) $$MR=\frac{M_{t}}{M_{i}}$$

### 2.7. Kinetic Model for the Anthocyanin Degradation

Fitting procedures (CurveExpert Professional 2.6.5) were used to determine the reaction rate constants ***k*** for the anthocyanin degradation. The general kinetic models of zero-, first-, and second-order reactions were used considering the Equations (11)–(13), respectively.

(11) $${\left[A\right]}_{0}+\left[A\right]=kt$$

(12) $$\left[A\right]={\left[A\right]}_{0}exp\left(-kt\right)$$

(13) $$\frac{1}{\left[A\right]}-\frac{1}{{\left[A\right]}_{0}}=kt$$

The half-life ($t_{1/2}$, h), which is the time required to achieve an anthocyanin degradation of 50% at a given temperature, was calculated by Equation (14) [25].

(14) $$t_{1/2}=\frac{Ln\left(2\right)}{k}$$

According to Fernández-Romero et al. [26], the effect of temperature was evaluated utilizing the Arrhenius equation.

(15) $$k=k_{0}\times e^{\left(-E_{a}/RT\right)}$$

The activation energy $E_{a}$ for each parameter’s reaction was determined by linear regression of Lnk versus 1/T by Equation (16) using CurveExpert Professional 2.6.5 software.

(16) $$Lnk=Lnk_{0}-E_{a}/RT$$

When $E_{a}$ (J/mol), R (8.3145 J/mol.K) is the universal gas constant, k is the reaction rate constant, and $k_{0}$ is the pre-exponential factor.

### 2.8. Anthocyanin Content Degradation

According to Fernández-Romero et al. [26] and Martins et al. [27], degradation was calculated according to Equation (17):

(17) $$A\%=\left(\frac{A_{i}-A_{t}}{A_{i}}\right)\times 100$$

where $A_{i}$ is the initial anthocyanin content at time 0 and $A_{t}$ is the anthocyanin content at time t.

### 2.9. Statistical Analysis

All results were expressed as mean ± standard deviation of four replicates for each experimental condition and observation evaluated. The quality of all the models’ experimental data was evaluated using the “Seed Water” package. The criteria for selecting the best fitting models were: the highest coefficient of correlation (*R*^2^) and the lowest standard error value (*RMSE*). All of the kinetic models for anthocyanin degradation were evaluated using ANOVA (*p* = 0.05).

## 3. Results

### 3.1. Kinetics Drying Process

Table 1 shows the mathematical models used by different authors to characterize the kinetic drying process in food. The logarithmic model best represents the blackberry bagasse drying process because it had a better fit, showing an *R*^2^ ranging from 0.956 to 0.959 and *RMSE* from 0.068 to 0.074 for all the temperatures studied. The analysis of variance carried out for each moisture value was significant (*p* < 0.05). Furthermore, at 90 °C, the Henderson and Pabis model has a good fit with an *RMSE* of 0.068 and *R*^2^ of 0.956. Contrary to this, at 80 °C, the Wang and Singh model had an *RMSE* of 0.081 and an *R*^2^ of 0.949. Likewise, the results show better values of *RMSE* and *R*^2^ as the drying temperature increases.

**Figure 1:**
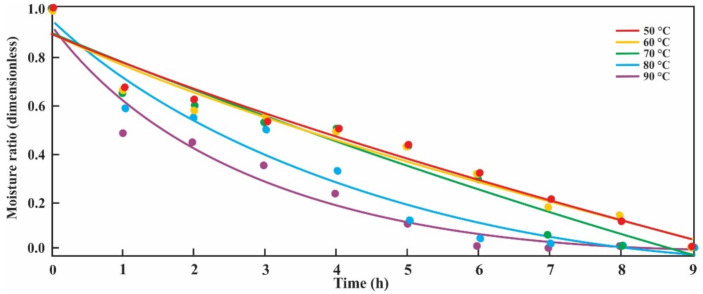
Logarithmic model fitted to the blackberry bagasse drying process. Dots represent experimental data, and solid lines represent predicted values.

Figure 1 shows *MR*’s experimental data versus time at different temperatures for the blackberry bagasse drying process. The solid lines correspond to the logarithmic model that describes the process, calculated by the “Seed Water” package. The curves show steep slopes at the 80 and 90 °C temperatures than at the other, lower temperatures. Blackberry bagasse reaches the equilibrium moisture content in 6 h at a drying temperature of 90 °C, while at 50 °C, a drying time of 9 h is needed. It can also be observed that at temperature values higher than 70 °C, a more significant decrease in the *MR* is seen.

### 3.2. Kinetics of Anthocyanin Degradation

**Figure 2:**
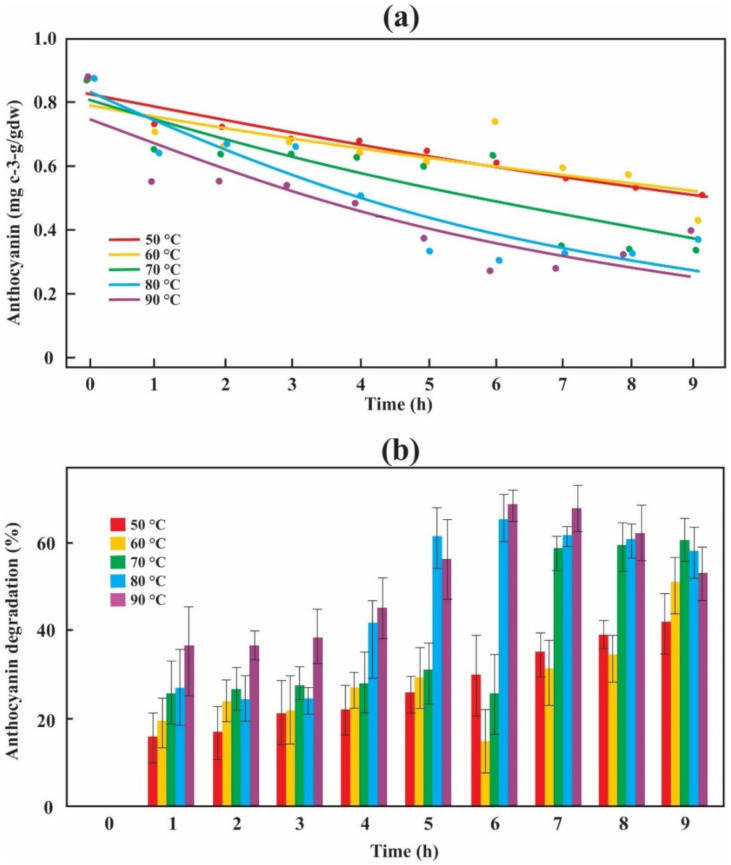
First-order kinetic model fitted to the kinetics of anthocyanin degradation during the drying process (**a**) and degradation changes of berry bagasse anthocyanin during the drying process (**b**).

The initial amount of anthocyanin at time zero was (0.88 ± 0.07 mg c-3-g/gdw). The CurveExpert Professional software results when plotting anthocyanin concentration data versus time were fitted to a first-order kinetic model between 50 and 90 °C (Figure 2a). At 50 °C, the anthocyanin content did not degrade considerably, reaching only 0.50 mg c-3-g/gdw from the 0.88 mg c-3-g/gdw initially. At 90 °C, the anthocyanin content’s most significant degradation was obtained, reaching up to 0.28 mg c-3-g/gdw. Figure 2b shows the evolution of anthocyanin degradation during the blackberry bagasse drying process. The most significant degradation (68.54%) occurred during the first 6 h of drying at 90 °C, and reached the stabilization phase later, while at 50 °C and 9 h, the degradation of anthocyanins reaches up to 42.56% and retention of 57.44%. The least degradation (15.62%) and the highest retention (84.38%) occurs in 1 h at 50 °C.

Table 2 summarizes the kinetic parameters calculated for each temperature evaluated. It can be noted that the *k* value increases as the drying temperature increases, these values range from 5.45 × 10^−2^ ± 4.68 × 10^−3^ to 1.21 × 10^−1^ ± 2.31 × 10^−2^ h^−1^. Further, Table 2 shows that 12.72 h is the half-life time required to achieve an anthocyanin degradation of 50% at 50 °C and 5.73 h at 90 °C. This parameter decreases as the temperature increases.

**Figure 3:**
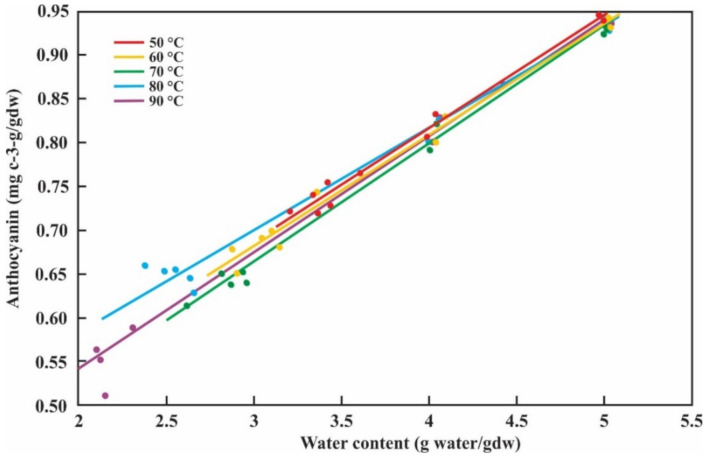
Moisture content and anthocyanin content during the blackberry drying process.

Figure 3 shows that, at any evaluated temperature, the anthocyanin content increases proportionally in a direct relationship with the moisture content (both on a dry basis).

### 3.3. Total Phenolic Content and Antioxidant Capacity

**Figure 4:**
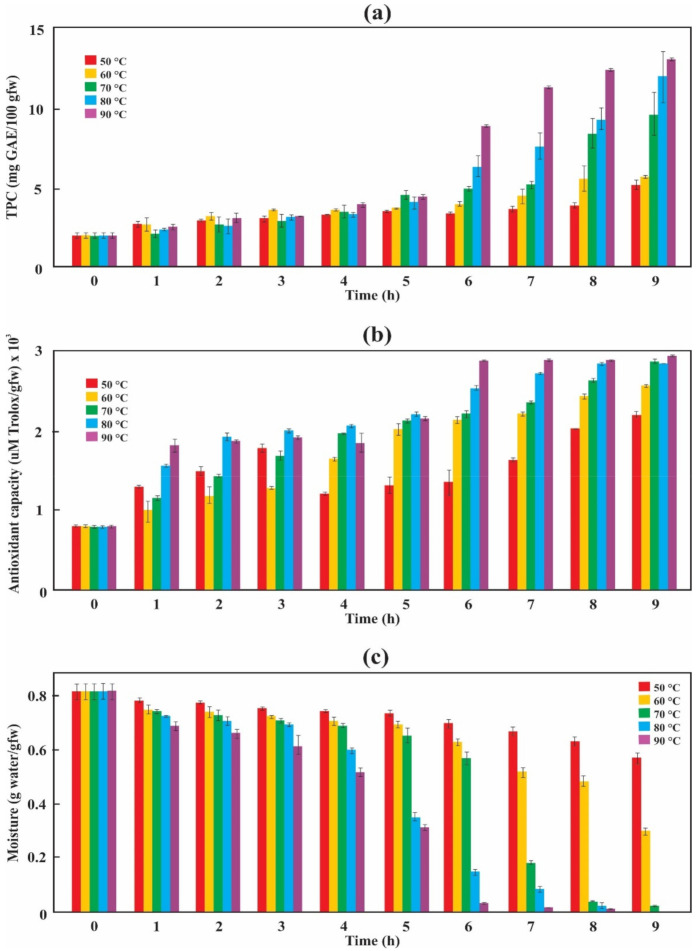
Changes in TPC (**a**), AC (**b**), and moisture (**c**) of blackberry bagasse during the drying process.

On a wet basis, the TPC values increase as the drying time increases (Figure 4a), reaching its maximum value (12.61 ± 0.05 mg GAE/100 gfw) at 90 °C in 9 h. A similar trend to TPC can be seen in the AC (Figure 4b) of blackberry bagasse, showing that a significant increase in its AC (2877.55 ± 3.76 µM Trolox/gfw) occurs at 90 °C up to 6 h of drying time and then stabilizes; while for the other temperatures, the AC increases up to 9 h of drying. The opposite occurs with the moisture content since it decreases as the drying time and temperature increase (Figure 4c). A sharp reduction in moisture content occurs at 90 °C after 6 h; this phenomenon is related to Figure 4a,b.

## 4. Discussion

Mathematical models for drying various berries, such as Lewis model, Page model, Henderson–Pabis model, Wang–Singh model, logarithmic model, Midilli–Kucuk model, and Weibull model [28], were reported. According to Table 1, the logarithmic model best represents the blackberry bagasse drying process from the Amazon region. In other studies, Martín-Gómez et al. [10] studied the blueberry convective drying-process. The Page model was the best one for the drying processes at 50 °C. Ross et al. [8] reported that the Page model fit the berries’ experimental data drying kinetics. Moreover, López-Vidaña et al. [12] show that the Midilli–Kuçuk model provides the best fit to the experimental data of the solar drying of blackberry because it presented the highest values of *R*^2^ (0.993 and 0.997). Based on our results, we can affirm that the logarithmic model can be used to predict the drying process of blackberry bagasse, and the drying process could be better described when working with temperatures above 70 °C. With the logarithmic model fitted to the drying process, we can see that at temperature values higher than 70 °C, a significant decrease in the *MR* is seen at the beginning of the process, according to that mentioned by Martín-Gómez et al. [10] and Rodríguez et al. [29]; then, the increment in temperature favors the rate of water elimination of the blackberry bagasse. According to Singh and Heldman [15], at equilibrium, all properties of a system will have fixed values; then, considering the logarithmic model, the equilibrium moisture is reached 6 h after starting the process with a temperature of 90 °C.

The anthocyanin contributes to the color appearance of fruits and vegetables and has a wide range of health-related benefits. They are also unstable and quickly degraded under thermal treatments [30]. Therefore, we consider it essential to know its degradation during the drying process. Anthocyanin extraction conditions can vary their content in different types of blackberries. Elisia et al. [31] reported 17.1 ± 0.9 mg c-3-g/g of crude blackberry extract. Contrary to us, the low content of anthocyanin (0.16 ± 0.04 mg c-3-g/gfw) in the blackberry bagasse used in the present study may be due to the solvent used in the extraction. As reported by Coklar and Akbulut [32], who obtained higher anthocyanin extraction performance when using methanol as solvent compared to water in *Mahonia aquifolium* berry, the same behavior was established by Yuan et al. [33]. Kinetic modeling is a helpful tool to predict the effect of the thermal process on anthocyanins’ degradation [34]. The blackberry bagasse drying process was carried out to analyze the degradation of anthocyanins by evaluating the kinetic models shown in Equations (11)–(13). The results of Figure 2a coincide with what was reported by Costa et al. [13], Mercali et al. [25], Chen et al. [35], and Qiu et al. [36]. This report suggests that the first-order kinetic model is suitable for predicting anthocyanin degradation of the blackberry bagasse when subjected to drying temperatures between 50 and 90 °C. Méndez-Lagunas et al. [16] carried out a process of the convective drying of strawberries at 50 and 60 °C and 1.5 m^2^/s of airspeed. They reported that degradation of anthocyanins of up to 45% was obtained in the first 100 min (1.67 h) and then a period stabilization until equilibrium is reached. López-Ortiz et al. [37] carried out a tray drying process of strawberries. They reported that temperatures of 40 °C and 60 °C resulted in a decrease in the anthocyanin concentrations of 68.92% and 71.32%, respectively. Our results show that up to 9 h of drying at 50 °C, anthocyanin degradation of 42.56% (57.44% retention) was observed, values lower than those obtained by the abovementioned authors (Figure 2b), while at 90 °C and 6 h the greatest degradation was observed. These results are related to the bioactivity of anthocyanins in the foods that contain them, which will be more affected by a high heat treatment than by a lower one [29,35].

In the work of Costa et al. [13], the thermal degradation of anthocyanin from açai pulp between 40 °C and 80 °C was studied, reporting a *k* value from 4.04 × 10^−4^ to 1.08 × 10^−3^ min^−1^. Qiu et al. [36] investigated the degradation kinetics of anthocyanins in purple potato slices subjected to air-impingement jet drying at 50, 65, and 80 °C, reporting a *k* value from 0.67 × 10^2^ to 1.07 × 10^2^ min^−1^. In both works, it was shown that at higher temperatures, the *k* values are also higher, and therefore there will be a faster degradation. These results coincide with ours reported in Table 2. The $t_{1/2}$ value (12.72 h at 50 °C) for the drying performed on blackberry bagasse is lower than the value found for the red raspberry juice storage at 37 °C (220.8 h) [35] and greater than the value found by Costa et al. [13] for acai pulp (25.8 h) at 50 °C; this difference might be due to the different anthocyanin compositions [35,37]. Applying the Arrhenius equation, we obtained an *Ea* value of 25.11 kJ/mol, values different from those obtained by Costa et al. [13] (24.16 kJ/mol) and Qiu et al. [36] (72.18 kJ/mol). When drying is carried out, the anthocyanin content on a dry basis increases directly proportional to the solid content (Figure 3). This is because when the drying temperature increases, there is greater evaporation of water similar results were obtained by López-Ortiz et al. [37].

When the drying data are expressed on a wet basis, we can see that at the beginning of the process (*t* = 0), the TPC was 1.92 ± 0.04 mg GAE/100 gfw (Figure 4a). These values are lower than those found by de Souza et al. [38] (850.52 ± 4.77 mg GAE/100 gfw) and Diaconeasa et al. [39] (260.74 GAE mg GAE/100 gfw). However, due to the sample’s concentration effect by water evaporation [22], the TPC values increase as the drying temperature increases. The behavior of the TPC coincides with the results reported by Martín-Gómez et al. [22] but differs from the results reported by Tan et al. [40] and Zielinska and Michalska [41] because they expressed their results on a dry basis. A significant property of anthocyanins is their AC, which plays an essential role in preventing neuronal and cardiovascular illnesses, cancer, and diabetes, among others [6]. Like TPC, the AC has the same tendency (Figure 4b), showing that a significant increase in its values occurs at 90 °C during the first 6 h of the process and then stabilizes in 2877.55 ± 3.76 µM Trolox/gfw. This value is higher than that found by Sariburun et al. [42] (90.95 ± 1.04 to 123.39 ± 1.02 µM Trolox/gfw). Ross et al. [8] state that convective drying at 70 °C maximizes the retention of the berry pomace anthocyanins’ bioactivity. The results observed in Figure 4a,b are related to Figure 4c; observing that, in general, both TPC and AC increase as the moisture content decreases during the drying process. Thus, high temperatures and extended drying times increase TPC and AC due to the water’s evaporation. This behavior occurs when expressing the data on a wet basis.

## 5. Conclusions

The blackberry bagasse drying process from the Amazon-Peru region is characterized by a logarithmic mathematical model, in which it can be predicted that the equilibrium moisture will be reached after 6 h of processing at 90 °C. During the drying process, a degradation of the initial anthocyanin content occurs following a first-order kinetic model. It is shown that a drying temperature of 90 °C is not suitable for blackberry bagasse because it causes a reduction in the anthocyanin content of up to 68.54% in 6 h. A direct relationship between anthocyanin content and moisture can be observed due to the phenomenon of water evaporation, which is also responsible for the increase in the TPC content and AC, calculated on a wet basis.

## Figures and Tables

**Table 1:** Kinetic models adjusted to the drying process of blackberry bagasse.

Model	Drying Temperature (°C)	Constants	*RMSE*	*R^2^*
Page	50	*k* = 0.224; *n* = 0.988	0.086	0.925
	60	*k* = 0.249; *n* = 0.938	0.088	0.919
	70	*k* = 0.223; *n* = 1.066	0.111	0.889
	80	*k* = 0.314; *n* = 1.082	0.090	0.938
	90	*k* = 0.537; *n* = 0.854	0.076	0.959
Henderson and Pabis	50	*k* = 0.211; *a* = 0.964	0.085	0.928
	60	*k* = 0.214; *a* = 0.953	0.087	0.923
	70	*k* = 0.239; *a* = 0.970	0.114	0.889
	80	*k* = 0.346; *a* = 0.984	0.090	0.937
	90	*k* = 0.434; *a* = 0.953	0.068	0.956
Newton	50	*k* = 0.220	0.081	0.925
	60	*k* = 0.226	0.084	0.918
	70	*k* = 0.247	0.108	0.888
	80	*k* = 0.351	0.085	0.936
	90	*k* = 0.454	0.069	0.953
Logarithmic	50	*k* = 0.047; *a* = 2.519; *c* = −1.623	0.068	0.959
	60	*k* = 0.061; *a* = 2.025; *c* = −1.134	0.075	0.949
	70	*k* = 0.042; *a* = 2.956; *c* = −2.064	0.091	0.938
	80	*k* = 0.231; *a* = 1.129; *c* = −0.181	0.082	0.955
	90	*k* = 0.378; *a* = 0.985; *c* = −0.045	0.074	0.956
Wang and Singh	50	*a* = −0.158; *b* = 0.006	0.081	0.934
	60	*a* = −0.164; *b* = 0.007	0.088	0.920
	70	*a* = −0.167; *b* = 0.006	0.098	0.916
	80	*a* = −0.244; *b* = 0.015	0.081	0.949
	90	*a* = −0.287; *b* = 0.020	0.113	0.920

**Table 2:** Kinetics parameters of anthocyanins’ degradation during the blackberry bagasse drying process and comparison with the bibliography.

Temperature	Present Work				Data from Costa et al. [13] *	
	*k* (h^−1^)	*R* ^2^	Error	$t_{1/2}\;(h)$	*k* (min^−1^)	$t_{1/2}\;(h)$
50	5.45 × 10^−2^ ± 4.68 × 10^−3^	0.946	0.028	12.72	4.47 × 10^−4^	25.8
60	4.71 × 10^−2^ ± 1.25 × 10^−2^	0.648	0.073	14.72	6.95 × 10^−4^	16.6
70	8.54 × 10^−2^ ± 1.73 × 10^−2^	0.771	0.087	8.12	8.67 × 10^−4^	13.3
80	1.26 × 10^−1^ ± 1.89 × 10^−2^	0.861	0.079	5.50	1.08 × 10^−3^	10.7
90	1.21 × 10^−1^ ± 2.31 × 10^−2^	0.779	0.090	5.73	----	----

* Data obtained for acai pulp.

## Data Availability

The data presented in this study are available in supplementary material.

## Supplementary Materials

The following are available online at www.mdpi.com/xxx/s1.
