# Supplementary material for: Kinetics Drying of Blackberry Bagasse and Degradation of Anthocyanins and Bioactive Properties
Source: Antioxidants (Basel). 2021 Apr 1;10(4):548. doi: 10.3390/antiox10040548 (PMC8065409; doi:10.3390/antiox10040548)
Supplement: Supplementary file 1 [file antioxidants-10-00548-s001.pdf]

S1. Bioactive properties

Tme	Temperature	TPC	Anthocyanin	CA	Moisture	Degradation
t0	T50	1.9582	0.8019	813.0006	0.8007	0.0000000
t0	T50	1.9138	0.9362	819.0579	0.8339	0.0000000
t0	T50	1.8604	0.9458	782.7145	0.8333	0.0000000
t0	T50	1.9493	0.8311	806.9434	0.8025	0.0000000
t1	T50	2.5360	0.7247	1323.5116	0.7765	9.6227100
t1	T50	2.5627	0.7617	1291.5993	0.7845	18.6414024
t1	T50	2.7493	0.7142	1316.1472	0.7732	24.4853954
t1	T50	2.7049	0.7500	1330.8760	0.7754	9.7518395
t2	T50	2.9093	0.7363	1488.5267	0.7712	8.1848894
t2	T50	2.8204	0.7147	1443.3151	0.7643	23.6564842
t2	T50	2.9004	0.7239	1480.9915	0.7673	23.4560343
t2	T50	2.8916	0.7353	1528.7148	0.7709	11.5245009
t3	T50	2.9360	0.6975	1825.2302	0.7554	13.0146295
t3	T50	3.1138	0.6749	1794.4641	0.7472	27.9079861
t3	T50	3.1493	0.6955	1750.5125	0.7547	26.4568407
t3	T50	3.1049	0.6787	1796.6617	0.7518	18.3330506
t4	T50	3.1938	0.6816	1186.3712	0.7465	15.0061304
t4	T50	3.2116	0.6833	1179.8235	0.7503	27.0129711
t4	T50	3.2382	0.6705	1196.1927	0.7423	29.1094561
t4	T50	3.2560	0.6843	1170.0019	0.7475	17.6654508
t5	T50	3.3182	0.6343	1241.9447	0.7276	20.9032821
t5	T50	3.3538	0.6806	1224.4306	0.7398	27.2984456
t5	T50	3.3804	0.6460	1347.0294	0.7292	31.6955343
t5	T50	3.3538	0.6329	1340.0238	0.7236	23.8446378
t6	T50	3.3982	0.6053	1440.2277	0.7003	24.5137029
t6	T50	3.4604	0.5839	1279.5212	0.6893	37.6300826
t6	T50	3.3893	0.6337	1260.3895	0.7103	32.9987091
t6	T50	3.4693	0.6293	1447.8804	0.7014	24.2848204
t7	T50	3.7716	0.5707	1641.6984	0.667	28.8272056
t7	T50	3.6560	0.5975	1638.1323	0.6783	36.1771664
t7	T50	3.5938	0.5500	1609.6035	0.6505	41.8492171
t7	T50	3.5404	0.5737	1620.3018	0.6687	30.9736763
t8	T50	3.9049	0.5312	2012.5890	0.6341	33.7546073
t8	T50	3.8071	0.5275	1997.7899	0.6233	43.6583783
t8	T50	3.8160	0.5185	2016.2888	0.6168	45.1754511
t8	T50	4.0204	0.5448	2008.8893	0.6353	34.4450377
t9	T50	5.7004	0.5065	2230.2725	0.5864	36.8364133
t9	T50	6.0471	0.5020	2188.4134	0.5784	46.3755798
t9	T50	5.9138	0.5086	2244.2256	0.5669	46.2195134
t9	T50	5.8693	0.4921	2202.3665	0.5611	40.7909726
t0	T60	1.9582	0.8019	813.0006	0.8007	0.0000000
t0	T60	1.9138	0.9362	819.0579	0.8339	0.0000000
t0	T60	1.8604	0.9458	782.7145	0.8333	0.0000000
t0	T60	1.9493	0.8311	806.9434	0.8025	0.0000000
t1	T60	2.6960	0.6835	959.3837	0.7567	14.7637773
t1	T60	2.5538	0.7476	930.7024	0.7689	20.1403914
t1	T60	2.5893	0.7006	997.6253	0.7534	25.9185193
t1	T60	2.9982	0.6947	1121.9107	0.7513	16.4074239
t2	T60	3.0338	0.6507	1133.4799	0.7411	18.8594156
t2	T60	3.0338	0.6801	1267.3164	0.7396	27.3542840
t2	T60	3.0960	0.7047	1254.2591	0.7487	25.4866475

t2	T60	3.3182	0.6404	1113.8941	0.7167	22.9475914
t3	T60	3.5049	0.6767	1283.6063	0.7287	15.6189916
t3	T60	3.3360	0.6892	1309.5442	0.7305	26.3832200
t3	T60	3.4604	0.6791	1286.8486	0.7265	28.1951366
t3	T60	3.4338	0.6708	1283.6063	0.7231	19.2896923
t4	T60	3.5493	0.6278	1650.2937	0.7076	21.7080453
t4	T60	3.6027	0.6240	1677.5267	0.6954	33.3515722
t4	T60	3.5316	0.6305	1671.4749	0.7054	33.3379832
t4	T60	3.5316	0.6860	1644.2419	0.7198	17.4579248
t5	T60	3.6827	0.6280	1999.8166	0.6905	21.6827490
t5	T60	3.6116	0.6384	1968.9737	0.6854	31.8036131
t5	T60	3.6560	0.6189	2067.6709	0.6894	34.5663277
t5	T60	3.6649	0.6018	2046.0809	0.6734	27.5933058
t6	T60	3.7449	0.7390	2180.8998	0.6201	7.8384781
t6	T60	3.8604	0.7807	2148.2336	0.6376	16.6087963
t6	T60	3.9138	0.7477	2139.3247	0.6245	20.9419404
t6	T60	3.8871	0.6939	2124.4764	0.6203	16.5026406
t7	T60	4.1716	0.5752	2213.1528	0.5119	28.2684651
t7	T60	4.7227	0.6314	2181.0547	0.5348	32.5570842
t7	T60	4.4827	0.5993	2189.8087	0.5207	36.6339039
t7	T60	4.7049	0.5892	2210.2348	0.5198	29.1071155
t8	T60	5.5938	0.5692	2444.9093	0.4954	29.0128119
t8	T60	5.3182	0.5658	2422.5566	0.4809	39.5599221
t8	T60	5.3004	0.5973	2447.7034	0.4938	36.8434156
t8	T60	6.2871	0.5885	2425.3507	0.4752	29.1898167
t9	T60	6.5538	0.4458	2548.1297	0.2975	44.4099259
t9	T60	6.6071	0.4251	2565.1926	0.2785	54.5965196
t9	T60	6.5538	0.4194	2576.5679	0.2894	55.6527573
t9	T60	6.5982	0.4265	2562.3488	0.2962	48.6761715
t0	T70	1.9582	0.8019	813.0006	0.8007	0.0000000
t0	T70	1.9138	0.9362	819.0579	0.8339	0.0000000
t0	T70	1.8604	0.9458	782.7145	0.8333	0.0000000
t0	T70	1.9493	0.8311	806.9434	0.8025	0.0000000
t1	T70	2.1716	0.6507	1161.9590	0.7411	18.8594156
t1	T70	1.9493	0.6535	1156.0873	0.7389	30.1996893
t1	T70	2.1004	0.6475	1182.5101	0.7465	31.5381913
t1	T70	1.9938	0.6563	1188.3819	0.7466	21.0344577
t2	T70	2.3138	0.6600	1432.8106	0.7382	17.7007412
t2	T70	2.2338	0.6258	1441.5419	0.7239	33.1562638
t2	T70	2.7316	0.6721	1456.0939	0.7365	28.9366016
t2	T70	2.7138	0.6115	1438.6315	0.7104	26.4184719
t3	T70	3.2560	0.6089	1652.8960	0.7056	24.0695689
t3	T70	3.0693	0.6249	1734.6001	0.7097	33.2472155
t3	T70	2.7493	0.6414	1699.5840	0.7104	32.1870506
t3	T70	2.8738	0.6776	1696.6660	0.7195	18.4726522
t4	T70	3.3271	0.6404	1978.6308	0.6965	20.1344673
t4	T70	3.2916	0.6340	1975.7379	0.6934	32.2814742
t4	T70	3.7982	0.6370	1978.6308	0.6813	32.6467542
t4	T70	3.4071	0.6248	1972.8451	0.6889	24.8210932
t5	T70	4.6071	0.6393	2107.7932	0.6723	20.2793425
t5	T70	4.5271	0.5878	2122.2326	0.6436	37.2127915
t5	T70	4.2427	0.6200	2125.1204	0.6656	34.4432064
t5	T70	4.5271	0.5704	2139.5598	0.6403	31.3664165
t6	T70	5.1227	0.7037	2185.8002	0.5857	12.2404446

t6	T70	5.0071	0.6516	2243.0648	0.5658	30.3984979
t6	T70	4.9271	0.6246	2245.9280	0.5436	33.9548341
t6	T70	4.9804	0.6016	2208.7060	0.5656	27.6149196
t7	T70	5.2916	0.3629	2375.4131	0.1906	54.7465924
t7	T70	5.0160	0.3572	2375.4131	0.1898	61.8451548
t7	T70	5.3093	0.3655	2349.7535	0.1904	61.3577143
t7	T70	5.1849	0.3581	2355.4557	0.1798	56.9103813
t8	T70	7.7360	0.3565	2670.8594	0.0368	55.5413543
t8	T70	8.5182	0.3507	2636.7624	0.0394	62.5355680
t8	T70	8.6249	0.3501	2656.6523	0.0376	62.9846616
t8	T70	8.9360	0.3442	2645.2867	0.0401	58.5791557
t9	T70	10.6071	0.3487	2869.2743	0.0089	56.5211463
t9	T70	11.5227	0.3460	2860.6626	0.0076	63.0386773
t9	T70	11.5227	0.3490	2903.7213	0.0098	63.1014075
t9	T70	10.1004	0.3468	2886.4978	0.0098	58.2719865
t0	T80	1.9582	0.8019	813.0006	0.8007	0.0000000
t0	T80	1.9138	0.9362	819.0579	0.8339	0.0000000
t0	T80	1.8604	0.9458	782.7145	0.8333	0.0000000
t0	T80	1.9493	0.8311	806.9434	0.8025	0.0000000
t1	T80	2.3138	0.6454	1542.9845	0.7256	19.5167835
t1	T80	2.3404	0.6547	1560.1492	0.7196	30.0676613
t1	T80	2.2960	0.6347	1571.5924	0.7278	32.8857709
t1	T80	2.3227	0.6320	1568.7316	0.7266	23.9594964
t2	T80	2.3849	0.6623	1957.0256	0.7065	17.4133247
t2	T80	2.6338	0.6607	1915.8007	0.7156	29.4289147
t2	T80	2.3760	0.7204	1948.7806	0.7212	23.8266740
t2	T80	2.8649	0.6414	1871.8275	0.7003	22.8285303
t3	T80	2.9449	0.6907	2000.7780	0.6998	13.8736338
t3	T80	3.1582	0.6453	1989.8828	0.6854	31.0703186
t3	T80	3.2116	0.6618	1984.4352	0.6965	30.0259529
t3	T80	3.1316	0.6434	1995.3304	0.6912	22.5780611
t4	T80	3.4693	0.5160	2051.4715	0.5898	35.6563048
t4	T80	3.3627	0.5226	2056.9502	0.6033	44.1727586
t4	T80	3.2293	0.4940	2078.8650	0.5934	47.7690032
t4	T80	3.2382	0.4935	2070.6470	0.5886	40.6231610
t5	T80	4.2782	0.3310	2207.7857	0.3345	58.7211404
t5	T80	3.9760	0.3331	2191.4430	0.3387	64.4173093
t5	T80	4.0738	0.3443	2166.9289	0.3476	63.5974232
t5	T80	3.7627	0.3323	2158.7576	0.3565	60.0202429
t6	T80	6.9182	0.3076	2547.6680	0.1575	61.6392654
t6	T80	6.5627	0.3021	2508.0435	0.1421	67.7312818
t6	T80	5.9493	0.3061	2535.2853	0.1463	67.6337169
t6	T80	6.2249	0.2955	2547.6680	0.1522	64.4435753
t7	T80	8.1004	0.3164	2707.9071	0.0717	60.5428115
t7	T80	7.8516	0.3358	2716.3314	0.0803	64.1303626
t7	T80	7.0604	0.3450	2713.5233	0.0798	63.5205867
t7	T80	8.0293	0.3319	2719.1395	0.0889	60.0685117
t8	T80	9.3360	0.3384	2829.3565	0.0109	57.7946919
t8	T80	9.1582	0.3389	2820.8178	0.0187	63.7960415
t8	T80	9.8960	0.3288	2849.2801	0.0279	65.2332966
t8	T80	8.9627	0.3334	2840.7414	0.0219	59.8812682
t9	T80	11.8604	0.3706	2940.7977	0.0035	53.7837838
t9	T80	11.1404	0.3643	2940.7977	0.0039	61.0915905
t9	T80	13.1493	0.3721	2938.1883	0.0018	60.6519132

t9	T80	11.4693	0.3636	2940.7977	0.002	56.2546145
t0	T90	1.9582	0.8019	813.0006	0.8007	0.0000000
t0	T90	1.9138	0.9362	819.0579	0.8339	0.0000000
t0	T90	1.8604	0.9458	782.7145	0.8333	0.0000000
t0	T90	1.9493	0.8311	806.9434	0.8025	0.0000000
t1	T90	2.5360	0.5874	1750.4476	0.6985	26.7509300
t1	T90	2.6782	0.5557	1794.1796	0.6813	40.6434125
t1	T90	2.5271	0.5639	1832.0807	0.6783	40.3732738
t1	T90	2.5360	0.5116	1872.8973	0.6834	38.4392559
t2	T90	3.1493	0.5578	1871.8227	0.667	30.4447691
t2	T90	2.8027	0.5639	1889.4072	0.6783	39.7627189
t2	T90	3.0427	0.5562	1874.7535	0.6505	41.1958375
t2	T90	3.0427	0.5388	1880.6150	0.6713	35.1712488
t3	T90	3.1938	0.5480	1926.3150	0.6256	31.6618301
t3	T90	3.2027	0.5688	1955.8547	0.6203	39.2429696
t3	T90	3.2027	0.5482	1917.4531	0.606	42.0415826
t3	T90	3.2027	0.5085	1935.1769	0.5795	38.8181363
t4	T90	3.7627	0.4752	1844.9971	0.5046	40.7421248
t4	T90	3.9404	0.4957	1767.0246	0.5207	47.0553121
t4	T90	3.7716	0.4775	1833.4456	0.516	49.5163025
t4	T90	3.7804	0.4768	1957.6240	0.5017	42.6339026
t5	T90	4.3849	0.3748	2142.7824	0.3086	53.2558303
t5	T90	4.3938	0.3788	2177.6167	0.3102	59.5331578
t5	T90	4.3849	0.3844	2148.5881	0.3202	59.3540808
t5	T90	4.5716	0.3793	2168.9081	0.3054	54.3565399
t6	T90	9.0071	0.2772	2879.0099	0.026	65.4357900
t6	T90	9.0160	0.2739	2876.0969	0.0302	70.7409603
t6	T90	8.9538	0.2725	2873.1840	0.025	71.1919916
t6	T90	8.9271	0.2760	2881.9228	0.0297	66.7899781
t7	T90	11.3538	0.2820	2886.1827	0.0045	64.8295709
t7	T90	11.3449	0.2824	2886.1827	0.0058	69.8347639
t7	T90	11.4427	0.2692	2897.7841	0.0052	71.5358219
t7	T90	11.3627	0.2908	2889.0831	0.0047	65.0131936
t8	T90	12.1804	0.3186	2903.9565	0.0036	60.2662829
t8	T90	12.2604	0.3143	2901.0309	0.0035	66.4318169
t8	T90	12.1893	0.3339	2901.0309	0.0038	64.6990069
t8	T90	12.1893	0.3469	2898.1052	0.0039	58.2582598
t9	T90	12.5804	0.4111	2956.8269	0.0019	48.7309672
t9	T90	12.5804	0.4306	2950.9808	0.002	53.9998052
t9	T90	12.6782	0.3743	2948.0577	0.0018	60.4231452
t9	T90	12.5893	0.3895	2953.9039	0.0018	53.1393350

S2. Moisture

Time	Moisure_50	Moisure_60	Moisure_70	Moisure_80	Moisure_90
0	4.52503061	4.52503061	4.52503061	4.52503061	4.52503061
1	3.494043747	3.128335488	2.895887118	2.63551561	2.180385637
2	3.318898875	2.802968897	2.67242273	2.461700001	2.003751825
3	3.037413339	2.666062709	2.46488814	2.260746755	1.555197668
4	2.94761404	2.416569808	2.227154783	1.462193927	

1.04447143
5 2.706244172 2.172770421 1.906983211 0.525401528
0.451683681
6 2.339004337 1.672106886 1.302470566 0.175868947
0.028521151
7 1.997782954 1.091803793 0.231034728 0.08721092
0.005075888
8 1.684800635 0.947292598 0.040016548 0.020291893
0.003713766
9 1.344267598 0.40940215 0.009108026 0.002808704
0.001878529
